# In Vivo Strain Patterns in the Achilles Tendon During Dynamic Activities: A Comprehensive Survey of the Literature

**DOI:** 10.1186/s40798-023-00604-5

**Published:** 2023-07-19

**Authors:** Naomi C. Adam, Colin R. Smith, Walter Herzog, Andrew A. Amis, Adamantios Arampatzis, William R. Taylor

**Affiliations:** 1 Institute for Biomechanics, ETH Zürich, Leopold-Ruzicka-Weg 4, 8093 Zurich, Switzerland; 2 Human Performance Laboratory, Faculty of Kinesiology, The University of Calgary, Calgary, Canada; 3 Department of Mechanical Engineering, Imperial College London, London, UK; 4 Department of Training and Movement Sciences, Humboldt‐Universität zu Berlin, and Berlin School of Movement Science, Berlin, Germany

**Keywords:** Achilles tendon, Tendon strain, In vivo measurement, Dynamic activities, Aponeurosis, Sub-tendon

## Abstract

Achilles’ tendon (AT) injuries such as ruptures and tendinopathies have experienced a dramatic rise in the mid- to older-aged population. Given that the AT plays a key role at all stages of locomotion, unsuccessful rehabilitation after injury often leads to long-term, deleterious health consequences. Understanding healthy in vivo strains as well as the complex muscle–tendon unit interactions will improve access to the underlying aetiology of injuries and how their functionality can be effectively restored post-injury. The goals of this survey of the literature with a systematic search were to provide a benchmark of healthy AT strains measured in vivo during functional activities and identify the sources of variability observed in the results. Two databases were searched, and all articles that provided measured in vivo peak strains or the change in strain with respect to time were included. In total, 107 articles that reported subjects over the age of 18 years with no prior AT injury and measured while performing functional activities such as voluntary contractions, walking, running, jumping, or jump landing were included in this review. In general, unclear anatomical definitions of the sub-tendon and aponeurosis structures have led to considerable confusion in the literature. MRI, ultrasound, and motion capture were the predominant approaches, sometimes coupled with modelling. The measured peak strains increased from 4% to over 10% from contractions, to walking, running, and jumping, in that order. Importantly, measured AT strains were heavily dependent on measurement location, measurement method, measurement protocol, individual AT geometry, and mechanical properties, as well as instantaneous kinematics and kinetics of the studied activity. Through a comprehensive review of approaches and results, this survey of the literature therefore converges to a united terminology of the structures and their common underlying characteristics and presents the state-of-knowledge on their functional strain patterns.

**Supplementary Information:**

The online version contains supplementary material available at 10.1186/s40798-023-00604-5.

## Key Points

- This comprehensive review identifies the inconsistencies of anatomical definitions of Achilles tendon sub-structures and provides guidelines to standardize definitions and measurements.
- The 107 included articles have mostly studied voluntary contractions as an activity and the medial gastrocnemius sub-tendon structure, but strains in the other sub-structures and during functional activities remain less clear.
- Critically, we demonstrate that the large range of strain results originates from unclear anatomical definitions, measurement methods, and activity-specific loading conditions.

## Introduction

The Achilles tendon (AT) is the largest tendon in the human body. It is a key passive mechanical structure that stores, transmits, and releases energy to enable upright standing and dynamic movements. It can withstand forces of up to 8–10 times bodyweight during sprinting [1] and perhaps consequently is also one of the most frequently injured tendons [2].

The most common pathologies are tendinopathy and rupture [3], which almost exclusively originate from exercise, as opposed to an underlying condition (2%) [3, 4]. Tendinopathies originate from chronic overloading, whereas ruptures usually take place after acute trauma [5]. Tendinopathy is characterized by pain and swelling that hinder movement [3] and that arise from excessive overloading from strenuous training regimens. The AT physiological response to frequent overloading involves collagen degeneration and sheath inflammation [6]. The main aetiology of AT ruptures, on the other hand, is an acute and high tension during a sudden movement [4], with or without prior degenerative changes. Indeed, some AT rupture sites show poor vascular supply or disorganized collagen fibres [7]. In that case, a sedentary lifestyle is often thought to cause these changes [8]. AT disorders can occur across the lifespan, but both injuries are most prevalent in middle-aged recreational and competitive male athletes [9]. Tendinopathies make up about 60% of all AT-related disorders [3] and are often diagnosed in, for example, track and field, tennis, volleyball, and soccer athletes, extending up to around 8% of annual incidence in top-level runners [3, 10]. Although the typical patient profile for AT rupture is the middle-aged “weekend warrior”, ruptures also occur at the elite level during gymnastics, basketball, or American football [11]. The incidence of AT injuries has steadily increased over recent decades [3, 12], currently affecting 2 in every 1000 people, indicating that AT pathologies are an important societal healthcare issue.

Recovering from an AT rupture can take up to a year or more and costs some US$14,000 for operative and conservative treatments alone [13]. Furthermore, patients recovering from AT rupture and tendinopathies frequently experience a permanent reduction in functional task performance, specifically a reduction in heel rise height and plantarflexion torque [14–16], often accompanied by a variety of associated comorbidities and substantially reduced quality of life [17].

To address these issues, a comprehensive understanding of the in vivo mechanical function of the AT during dynamic activities could provide important insights into optimal physiological function of the tissue, the effects of training programs [28, 29], understanding injury mechanisms, and improving surgical repair techniques and rehabilitation protocols.

AT strain, the elongation of the tendon relative to its slack length provides a quantifiable metric to investigate dynamic AT function within healthy and pathologic populations. In vivo AT strain measurement enables an understanding of the role of the AT in triceps surae muscle tendon unit (MTU) function through energy storage, release, and dissipation. In vivo AT strain measurements have recently gained traction as the measurement methods have become more tailored to dynamic activities. Even though MRI had already been used to study tissue strain, strain gauges, ultrasound and motion capture coupled with modelling have all gained traction as tools for accessing in vivo strain. Studying the global AT behaviour has enabled important insights into in vivo MTU function, and knowledge of AT strain and force patterns has helped establish boundary conditions for modelling or guiding ex vivo experiments [18]. Studying AT behaviour also allows an estimation of energy storage and release during movement, and absorbing energy during landing [31], which are key interests in sport science. Since AT loading and strains are thought to be greatest during jumping and landing, these activities present a high risk of AT rupture.

Anatomical studies have demonstrated that the AT is comprised of three twisting sub-tendons originating from the gastrocnemii and soleus that exhibit non-uniform strain patterns [19]. While the anatomical definitions of these structures remain diverse, understanding their local AT strains during dynamic activities could have great implications. Firstly, differences in AT spatial strain uniformity are known to occur between healthy and pathologic populations [20], where characterizing local strain patterns might help track how MTU function adapts with pathology. Furthermore, in the case of AT ruptures, the tear almost always occurs at the same location, two to four centimetres above the calcaneal insertion [21]. Here, calcaneal valgus and varus malalignments are hypothesized to be factors that contribute to rupture during running, as they could induce extra shear loading (forces orthogonal to the long axis of the structure) on the AT fibres [3]. Since strain magnitude and strain rate (how fast the tendon stretches) are two known predictors of tendon failure [22], characterizing the AT local strain patterns in vivo could shed light on its associated injury mechanisms. Finally, strain applied to tendons in vitro has been shown to result in collagen adaptation [23] and tissue remodelling. Indeed, an applied strain range based around 6% has led to anabolic collagen adaptations in vitro in rats [24]. Similarly, in humans, in vivo plantarflexion training at 5% strain magnitude showed an increase in stiffness of the AT [25], but the extent to which such adaptation promotes the cascade of healing processes is not fully understood [26]. Knowledge of the relationships between global and local strain patterns could therefore enhance our understanding of anabolic remodelling at specific AT-injured sites. However, measuring non-uniform strain patterns in humans during in vivo activities remains challenging because of the inherent limitations of measurement methods and the complex AT architecture. While characterizing global and local strain patterns of the AT has a great fundamental and applied potential, identifying where error sources influence strain results is critically important.

This study therefore builds on previous summaries of AT tissue function [27, 28] to review in vivo measurements of the healthy human AT to provide the current state of knowledge regarding functional elongation patterns during dynamic activities. Thus, our goal is to (1) unify the anatomical definitions of the AT sub-structures to provide a common consensus, (2) review the peak values and their ranges, as well as temporal and spatial variability in AT strain patterns during functional activities, and (3) provide an overview of the key factors that govern AT strains. Based on our comprehensive survey of the literature, we provide best practices to improve the quality of AT strain measurements.

## Review Methods

### Rationale

The goal of this review was to provide an extensive understanding of the strain patterns experienced by the human Achilles tendon during dynamic activities in healthy populations. Observations made in the literature were based on several metrics, mostly elongation (difference between the tendon instantaneous length and a reference length) and strain (defined as $\varepsilon (\%)=\frac{L-{L}_{0}}{{L}_{0}} x 100$, where ${L}_{0}$ is the AT slack length and $L$ is its instantaneous length). However, one issue that makes quantification of strain in musculoskeletal structures (and particularly the AT) extremely difficult is determination of the slack length. As a result, ${L}_{0}$ is often assigned a reference length based on an arbitrarily selected pose or posture where the muscles would ideally be inactive. When the reference length is different from the slack length, the investigated strain actually becomes relative elongation. Additional characteristics of time-varying strain throughout different activities, such as tendon recoil (difference between the tendon's maximal and shortest length within an activity cycle), longitudinal, transverse, or antero-posterior displacements, are rarely reported but could add considerable understanding to the biomechanical characteristics of this structure. Overall, two types of valid measurements are found in the literature: strain results, which are normalized and can be used for comparison, and other metrics. We therefore included in our search two types of results based on metrics: quantitative results of strain, and qualitative results, which entailed insightful observations and other metrics. Additionally, dynamic activities of interest comprised varied movements either performed during daily activities or during exercise. These included voluntary contractions, walking, running, hopping, jumping, and landing. Voluntary contractions referred to isometric contractions whose force output was measured by a dynamometer and that were normalized to the maximal force exerted by the subject in a test trial. The voluntary contractions found in the literature ranged from 10% of the maximal exerted force to 100% (maximal voluntary contractions). Additionally, walking and running trials were included for all speeds, and for both overground and treadmill motion. Finally, several kinds of jumps or landings have been included. They entailed single- or double-legged hopping, squat, or countermovement jumping, as well as double-legged landings. The methods were also considered during the search and were required to contain at least one live or direct measurement of strain during movement. As a result, MRI, 2D or 3D ultrasound, as well as motion capture combined with modelling, was chosen. Measurement with strain gauges did not take place in human ATs and was therefore not included. Similarly, pure modelling, which did not entail live recording, was not selected.

### Systematic Search Method and Inclusion Criteria

The literature databases Scopus and PubMed were searched from database inception until 6 Dec 2021 using the EPPI-Reviewer software suite (EPPI-Reviewer4 v.4.12.2.0) based on the following search string:

“(Achilles tendon) AND (elong* OR length OR lengthening OR length change OR strain. OR strain rate OR in vivo)”. The studied populations were adults (> 18 years old), with no history of AT-related pathology. Articles were included if they reported in vivo measurements of human AT strain during dynamic activities such as walking, running, hopping, jumping, landing, or voluntary contractions and measured with ultrasound, MRI, or motion capture coupled with modelling.

**Fig. 1:**
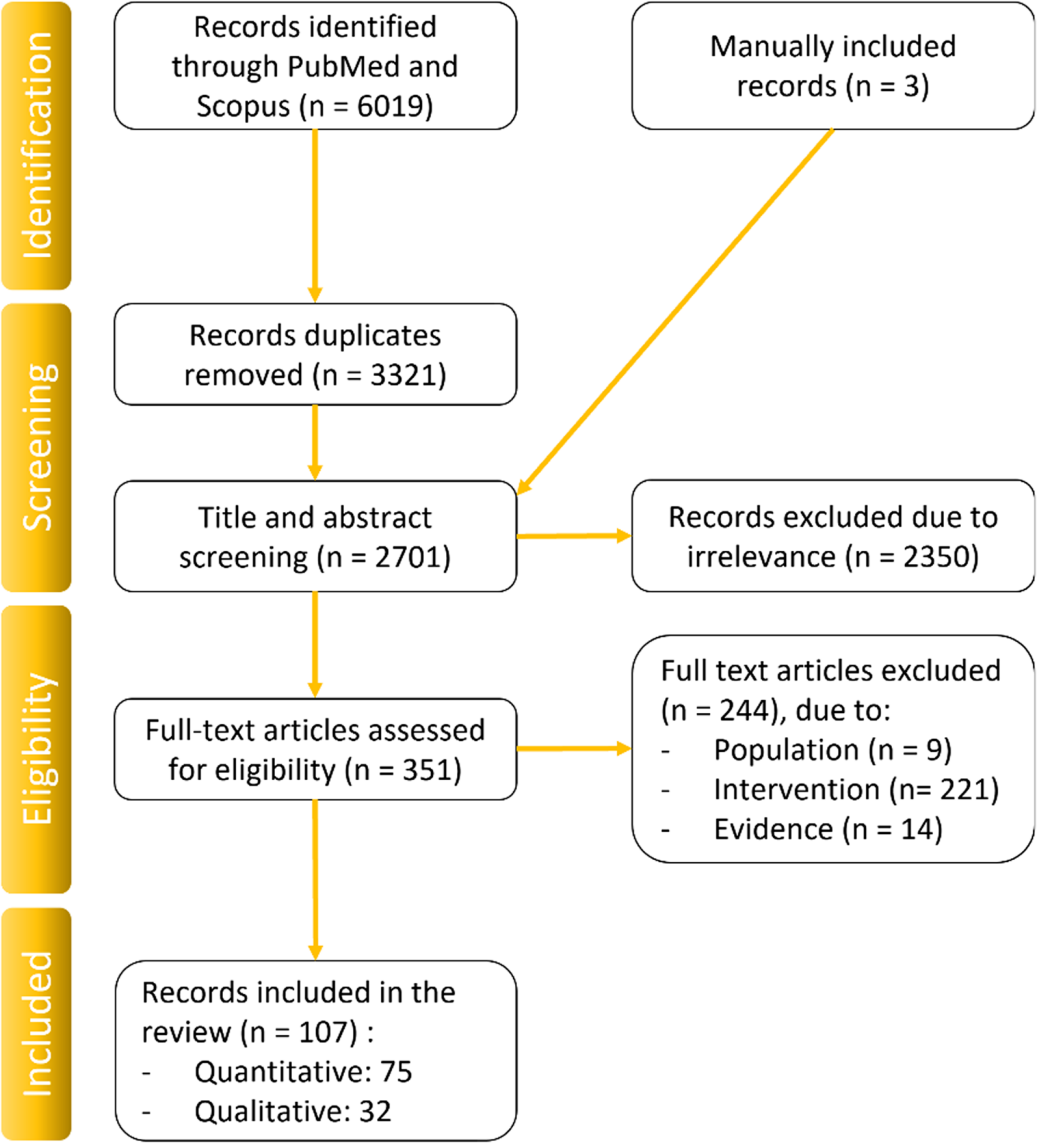
PRISMA diagram of the articles included in this review

Additionally, “non-strain” or qualitative articles, meaning articles providing metrics such as displacement or relative elongation, were added if they disclosed time-varying curves or indirect insights, such as elongations. Author NCA performed the preliminary screening for exclusion based on the articles´ abstracts, before reviewing the full texts. Any uncertainty about texts for inclusion was resolved with WRT and CRS. Three further records that met the previous criteria were found in the cited literature of the included studies and manually included. Finally, 107 articles were included in this review, which entailed 75 articles that were able to provide quantitative data on AT strain during functional activities (Fig. 1).

### Data Extraction and Collection

In this study, we extracted peak strain values (longitudinal and transversal) reported in the included literature, either from tables or from time-varying curves. When the strain values were extracted from plots, data points were digitized from figures using WebPlotDigitizer v4.2. We also extracted and included strain values found in the discussion sections. All elongation and displacement results (with or without provided reference lengths) are reported as qualitative results. Strain results where reference lengths, measurement locations, or methods were not thoroughly disclosed were also presented as qualitative results. In such cases, to provide an estimate of strain, we converted these displacement values assuming a reference length of AT and aponeuroses of 400 mm [29] (our estimated values are indicated with an asterisk in the tables). All the qualitative and quantitative results stored in tables, as well as the graphs of strains with respect to time of the dynamic activities, are to be found in the supplementary online resources. Additionally, the working principles behind each measurement method are summarized in the “Techniques for Strain Measurement” section (Additional file 1: Online Resource S1). Specifics pertaining to each measurement protocol such as the reference length definition (for strain results), the measurement location, and the activity details can be found in the results tables (Additional file 2: Online Resource S2).

## Results

### Achilles Tendon Anatomy

**Fig. 2:**
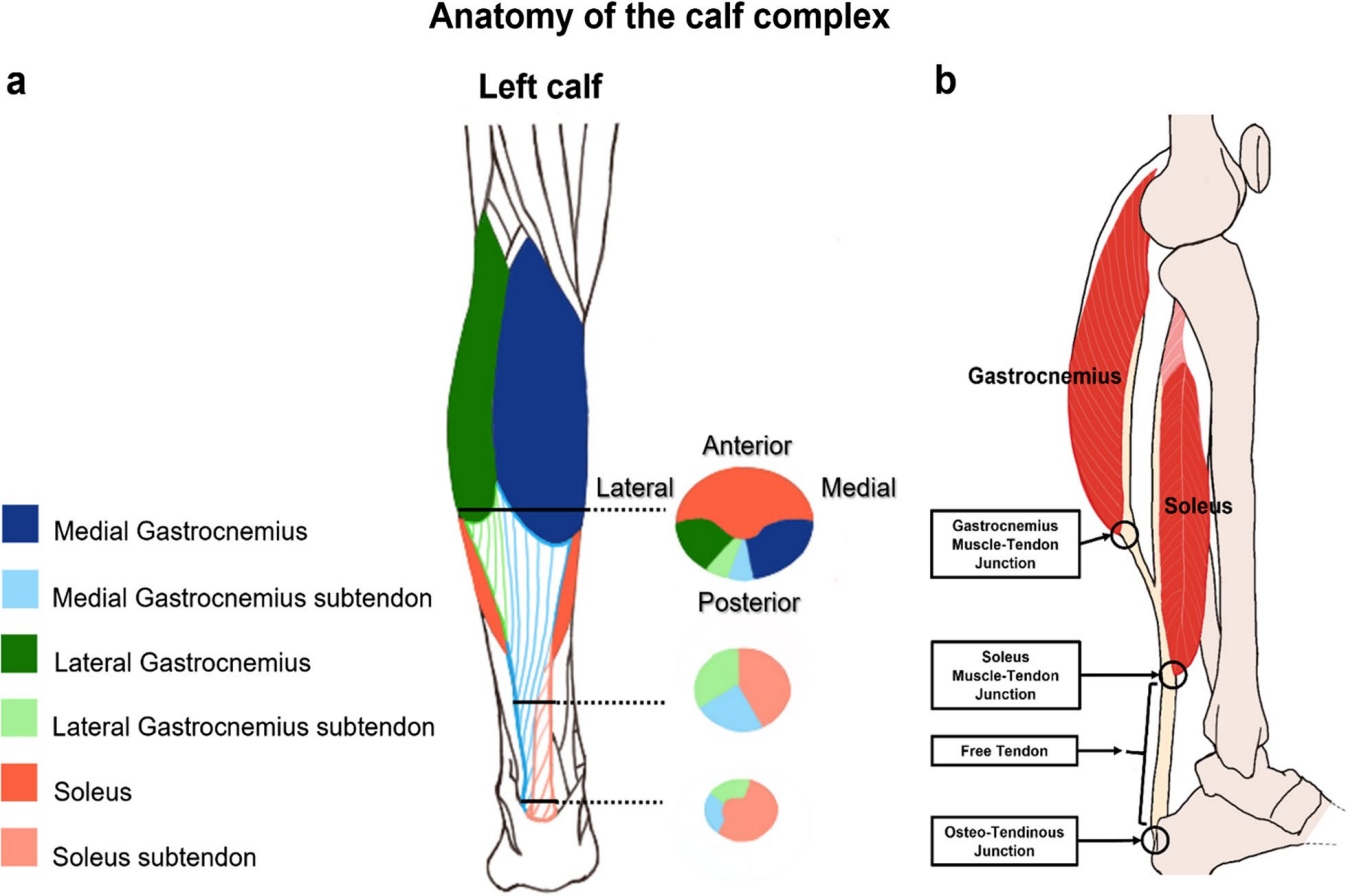
**a** Three axial cross sections of the AT sub-tendons demonstrating their natural twist [19], **b** sagittal cross section indicating common anatomic naming conventions of the triceps surae muscles and the Achilles tendon (note: spaces between soft tissues are exaggerated for graphical clarity)

**Fig. 3:**
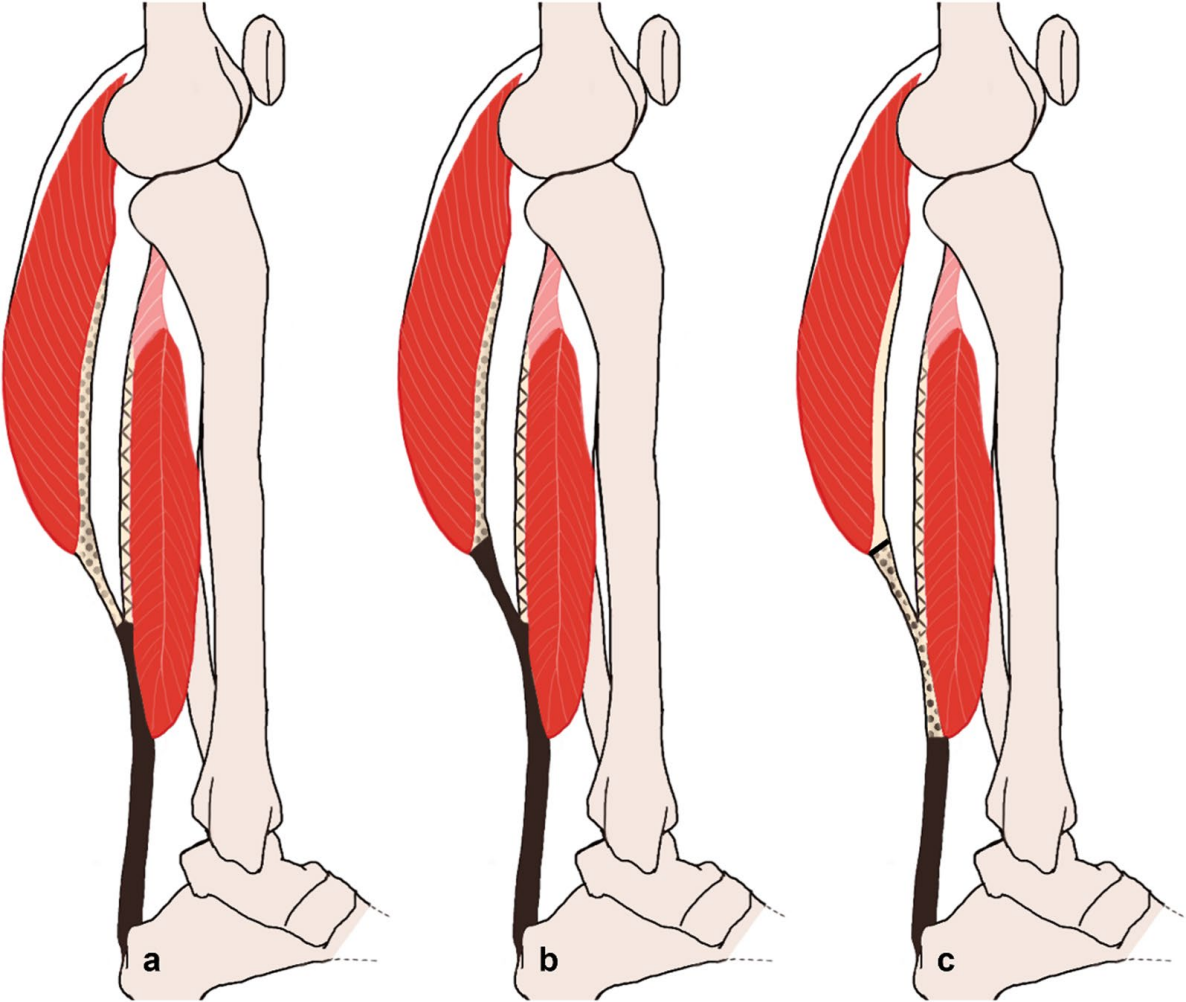
Three different definitions **a**–**c** of the gastrocnemii aponeuroses (dotted), soleus aponeurosis (lines), and Achilles/free tendon (solid black) are found in the literature; however, in this review convention “c” was chosen because it is most commonly used (note: spaces between soft tissues are exaggerated for graphical clarity)

Understanding the variation of anatomical definitions of the AT is important for interpreting published strain results. This section aims at presenting the basics of AT anatomy (Fig. 2), common measurement challenges, as well as different definitions found in the literature. Additionally, all strain values included in this review will be presented according to convention “c” (Fig. 3) in the results and discussion sections whenever possible.

Anatomically, the AT is composed of three mechanically separate sub-tendons that originate from the medial gastrocnemius (MG), lateral gastrocnemius (LG), and the soleus muscle–tendon junctions (MTJs), which all join and insert into the calcaneus at the osteo-tendinous junction [19] (Fig. 2, panel b). The entire AT tendon, from the gastrocnemii MTJ to the osteo-tendinous junction, is on average 150 mm long (varying from 110 to 260 mm) and its medial–lateral width tapers from some 7 mm (superior) to approximately 2 mm (inferior) [30]. Although the pure-tendinous region between the osteo-tendinous junction and the soleus MTJ has been named by a few authors as the “Achilles tendon”, common consensus now describes this region as the “free tendon” (FT) (Fig. 2, panel b).

Cadaveric studies show that the three sub-tendons rotate internally from proximal to distal leading to both gastrocnemius sub-tendons inserting into the calcaneus laterally, with the fibres originating from the soleus inserting medially [31] (Fig. 2, panel a). However, the magnitude of the twist varies from 10 to 150° across the population [32]. Moreover, the AT is the culmination of three MTUs, where each MTU consists of all the structures from a single triceps surae muscle to the distal osteo-tendinous junction. One of these structures is the aponeurosis, which links the tendon to the muscles and is defined controversially in the literature. The formal anatomical definition of the aponeurosis was established in cadavers and describes the structure as the fibrous tissue proximal to the point where the gastrocnemius MTUs and soleus separate [19] (Fig. 3a). However, because this point is difficult to identify in in vivo images, some authors chose to define the gastrocnemius aponeurosis as only the fibrous intra-muscular tissue proximal to the gastrocnemius MTJ (Fig. 3b). Since the majority of AT research literature is based on ultrasound techniques that most easily locate the soleus and gastrocnemius MTJs, however, the most commonly used definition of the aponeurosis has recently become the fibrous tissue between the soleus and the gastrocnemius MTJs (Fig. 3c), even though this convention does not incorporate the proximal connective tissue along the MG muscle. Finally, for clarity, we refer to the three “sub-tendons” within this review as the structures joining the given MTJ (MG, LG, or soleus) and the calcaneus (osteo-tendinous junction), even though this definition does not necessarily account for the natural twist of the fibres from an anatomical perspective.

### AT Strain Patterns during Functional Activities

**Fig. 4:**
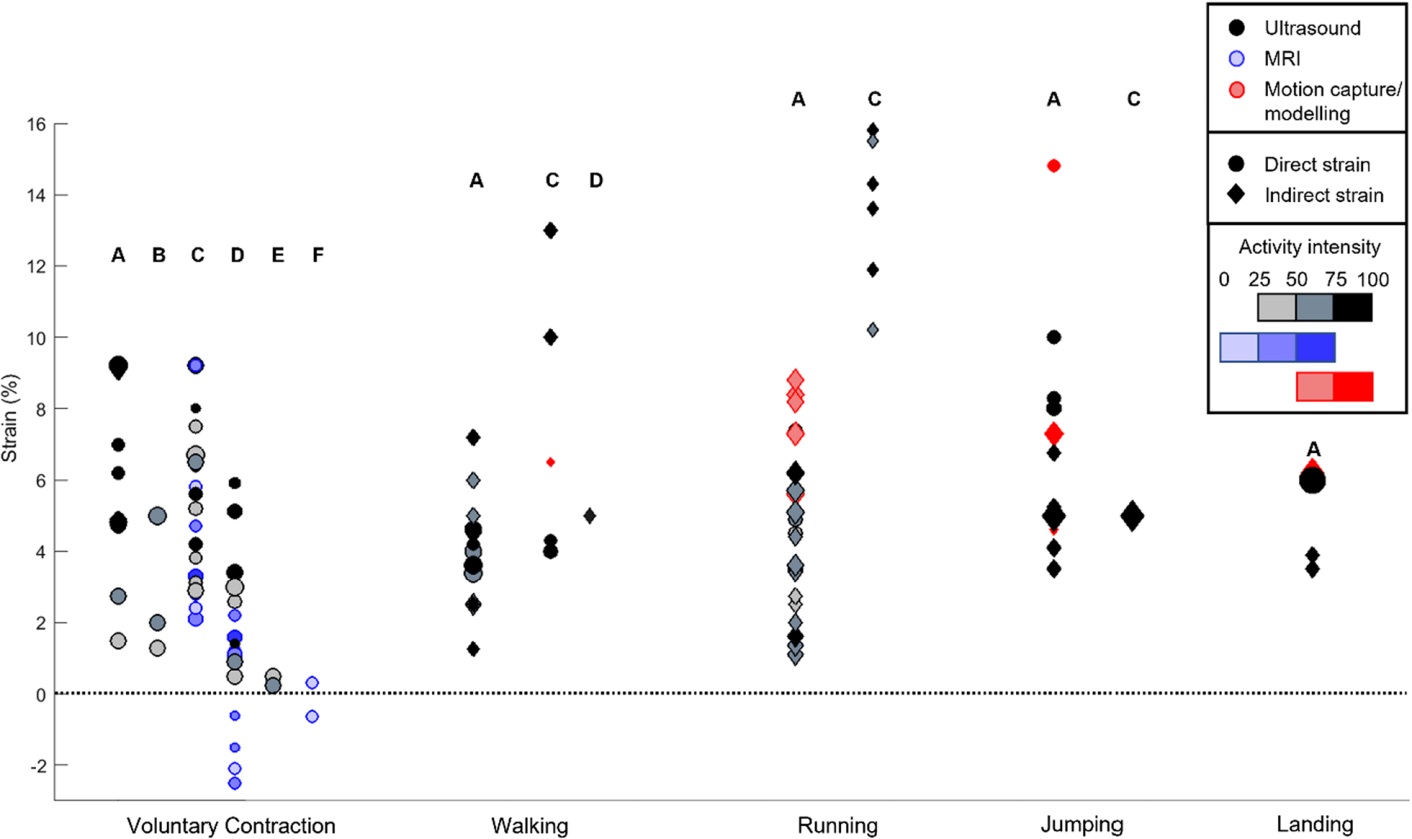
Peak strain results across activities: results are split by Achilles tendon location. The size of the data points is proportional to the number of participants. Colours indicate measurement methods; the filling gradient represents the speed or intensity of the activity and the shapes if the strain had been directly measured or was an indirectly obtained value. A: Medial gastrocnemius sub-tendon, B: lateral gastrocnemius sub-tendon, C: free tendon, D: medial gastrocnemius aponeurosis, E: lateral gastrocnemius aponeurosis, F: soleus aponeurosis

The quantitative strain results, as well qualitative observations, were sorted and reported by activity. The peak values and the strain ranges, for each sub-structure when possible, were disclosed to present the current overview of the literature (Fig. 4).

#### Isometric Contraction

Maximal voluntary contraction (MVC) or submaximal voluntary contraction (VC) was the most studied activities [25, 33–99], while the MG sub-tendon (free tendon plus MG aponeurosis) structure has been most investigated. The reported peak longitudinal strain values during VCs suggest the MG sub-tendon strains by up to approximately 9.2% (Fig. 4 and Additional file 2: Online Resource S2, table MVC). The MG sub-tendon generally strained more than the LG sub-tendon [74, 81]. However, the longitudinal strain distribution along the sub-tendons is not homogeneous. For example, in the MG sub-tendon the free tendon generally exhibits larger strains than the aponeurosis [63, 67, 83, 93] and two articles report the opposite trend [37, 99].

**Fig. 5:**
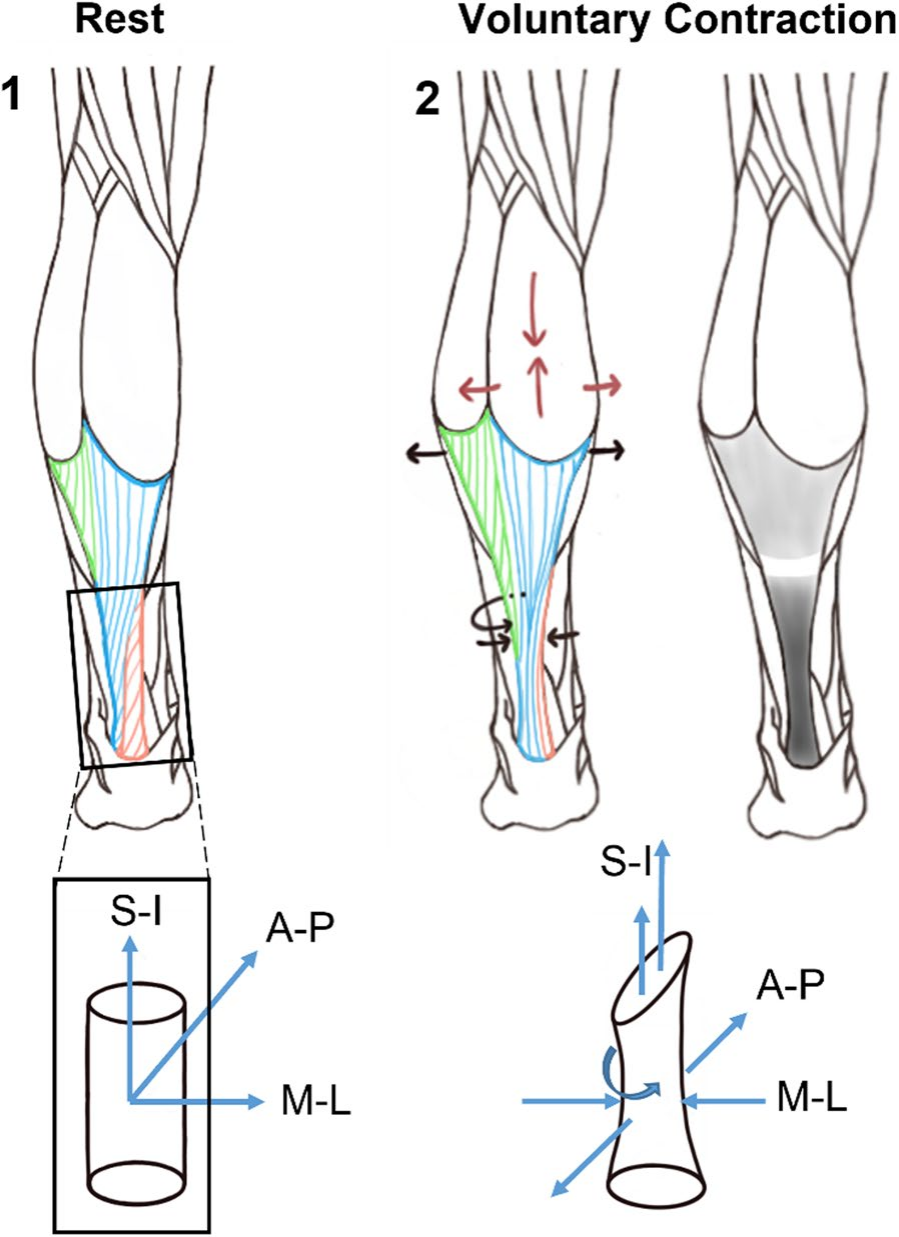
Strain patterns of the Achilles tendon during a voluntary contraction. The two cylinders represent a close-up of the free tendon at rest (panel 1) and during contraction (panel 2), assuming no change in knee and ankle joint angles. In panel 2, the red arrows indicate the shortening and bulging of the gastrocnemius muscle during contraction, while the black arrows illustrate the positive transversal strain (widening) around the gastrocnemius aponeurosis, as well as the negative transverse strain (thinning) at the free tendon. Additionally, the circular black arrow around the free tendon represents the rotation undergone during contraction. The grey scale scheme represents the magnitude of the longitudinal strain undergone by the tissue: the darker the colour, the greater the longitudinal strain. The white region around the soleus muscle tendon junction indicates no strain or even a negative longitudinal strain [86]. (S-I: superior–inferior, A-P: anterior–posterior, M-L: medio–lateral)

When the free tendon alone is considered, peak strains are reported to increase linearly with respect to activation during sub-maximal contractions (up to 70%) [63, 67]. Interestingly, the longitudinal elongation is not uniform in the anterior–posterior direction, as the mid- and deep regions of the FT displace more than the superficial (posterior) region [43, 74] (Fig. 5). The longitudinal strains in the FT with VCs are associated with either a small positive [87] or, more commonly, a negative transverse strain depending on the region [87, 92, 96] (Fig. 5, panel 2), resulting in a reduction of the cross-sectional area (average approximately 5.5%) of the free tendon [53, 68, 69]. The greatest changes were reported mid-structure [93], including a decrease of the medio-lateral width (approximately 9%) [68], but interestingly also a slight increase in the antero-posterior thickness [68, 69]. Finally, VCs result in an external rotation of the free tendon relative to the calcaneal insertion, especially in the mid-portion [68], where the rotation reduces the natural twist of the sub-tendons compared to their resting state.

In the aponeurosis, longitudinal peak strains were consistently smaller than in the FT during sub-maximal contractions, where most of the elongation is reported to occur during the first 25% of activation [63, 67], indicating a nonlinear (e.g. viscoelastic) response as opposed to the more linear behaviour of the FT. Furthermore, while the entire aponeurosis structure consistently showed a global positive longitudinal strain [57, 63, 67, 83, 86, 87, 90, 93, 100], a small shortening of the distal aponeurosis has been observed, around the interface with the soleus MTJ [57, 86, 100] (Fig. 5, panel 2). As a result, the strain distribution even within the aponeurosis is considered non-homogeneous. Here, transversal strains are discussed controversially: values between 5% [83] and 9% [87] M-L thickening have been reported for the MG aponeurosis, but an M-L thinning of this region has also been observed [96]. Finally, with regard to knee joint angle, the MG aponeurosis strained more than the soleus aponeurosis when the knee was extended, but the soleus aponeurosis extended more than that of the MG when the knee was flexed at 90° [97],

#### Walking

All studies assessing AT strain during walking relied on ultrasound measurement [29, 42, 101–110], with the exception of Yamamoto [111] and Pizzolato and co-workers [112], who used motion capture-based methods. Only five studies reported direct strain values [29, 105, 106, 110, 112]. Three definitions were used for the AT reference length: length at standing [101], heel strike [105], or toe-off [107].

During walking, the tendon is minimally loaded before heel strike (Additional file 1: Online Resource S1). Thereafter, the triceps surae ramp up their contraction throughout the stance phase, which allows energy to be stored in the AT, where the peak strain (only measured in the MG sub-tendon in the literature) reaches between 4% [101] and 4.6% [29] (Additional file 2: Online Resource S2, table walking). In the last 25% of the stance phase, the tendon recoils, and the foot is pushed off the ground. Only elongation data were available for the LG sub-tendon [103, 111], which was reportedly similar to the MG [111]. Furthermore, longitudinal elongation seemed to vary regionally within the LG sub-tendon, with higher strains in the free tendon than in the aponeurosis or the whole sub-tendon [103]. A more homogeneous strain distribution was observed in the MG and FT [105] than in the LG sub-tendon. Finally, it seems unclear whether the superficial FT displaces more [103] or less [104] than the deep layer.

#### Running

For studies addressing running [29, 79, 107, 113–123], the AT reference length definitions included: standing [113], heel strike [119], 20° ankle plantarflexion [106], or toe-off [120]. Strains were generally obtained using ultrasound [29, 79, 106, 107, 113, 117, 119, 120, 122, 124], but also using modelling approaches, deriving the strains from the calculated force [115].

[29, 106] (Additional file 1: Online Resource S1). The entire MG sub-tendon reaches a peak strain of upto 8.8% [123], whereas the free tendon is thought to be strained up to 15.8% [119] (Additional file 2: Online Resource S2, table running).

Regarding measurement methods, one model-based estimate of peak MG sub-tendon strain was in good agreement with ultrasound measurements: 8% [123]. However, studies that have modelled the free tendon alone resulted in similar estimates of about 8% [115] and 6% [75], but ultrasound measurements of this structure were as high as 16% [119]. Comparisons between forefoot and rearfoot running demonstrated that higher AT strains are found in forefoot running [116, 119, 125] at 10 km/h. Interestingly, this difference decreases with increasing speeds [119]. Finally, recoil values were measured for both loaded and unloaded running, as well as before and after a training program [79, 122]. Despite a similar peak strain magnitude, the recoil value was almost two times greater during loaded running (+ 20% body mass added). Additionally, after a 10-week plantarflexion training, an unexpected similar peak strain value was measured, but the tendon recoil diminished by 30% during the late stance phase.

#### Jumping and Landing

Several kinds of jumps or landings have been analysed: single- [113, 126, 127] or double-legged hopping [128–131], squat or countermovement jumping [132–135], as well as double-legged landings [133, 136, 137]. The only data available for jumping and landing are measurements of the MG sub-tendon, reaching a peak strain between 8 [113] and 15% [128] (Fig. 5, Additional file 2: Online Resource S2, table jumping and landing and Additional file 1: Online Resource S1). The AT strain then decreases throughout the remainder of the ground contact time as it releases energy. The reference length was defined at standing [129, 134], ground contact [128], at the length corresponding to 5% of the maximal measured ground reaction force [126] or taken from the literature [132]. Additionally, different assessment methods yielded different results: all modelling studies reported strains that were lower than those measured using ultrasound. Interestingly, AT strains decreased during double-legged hopping when the jumping frequency increased [130]. Indeed, the MG sub-tendon length change accounted for 90% of the total MTU length change at 2 Hz, whereas it plummeted to 52% at 3.5 Hz. The AT strain behaviour shows two phases during landing. First, shortly after initial contact, minimal lengthening is observed [137]. Second, the AT is then strained to between 3.5% [136] and 6% [133].

## Discussion and Consensus

The Achilles tendon is critical for transferring load, enabling movement of the lower limbs, and storing energy to allow highly dynamic and challenging functional activities to be performed. While the structure’s material properties, strain behaviour, and loading conditions have been relatively well characterised [22, 138], only limited consensus has been reached on its functional behaviour in vivo. In this comprehensive survey of the literature, we therefore aimed to understand the underlying reasons for the inhomogeneous outcomes and provide a clear overview and comparison of its in vivo anatomical and strain behaviour during various functional movements. To achieve this, in vivo strain data of the AT were systematically extracted and analysed from the existing literature. The complex twisted geometry of the AT, combined with its one- and two-joint origin attachments, and the variability in local strain patterns, as well as different definitions of the AT reference length across studies, confounded direct comparisons of strain magnitudes. In addition, the measurement techniques, including ultrasound, MRI, and movement analysis/modelling, each possess inherent limitations. Overall, the highest peak strains were reported during jump take-off and running, while jump landing, walking, and isometric contractions exhibited lower peak strains (Fig. 4). Regionally within the AT, the FT shows greater longitudinal strains than the aponeuroses [63, 67, 83, 93]. Negative transverse strains are consistently reported in the FT upon triceps surae contraction [87, 92, 96]. However, both positive and negative transverse [83, 87, 96] and longitudinal strains have been observed in the aponeuroses, indicating the need for further research to comprehensively understand their behaviour. Moreover, conflicting strain patterns have been observed in the FT between the deep and superficial layers, but it remains unclear whether this is a valid difference or whether it is due to methodological limitations.

### Impact of Measurement Methods and Protocols on Strain Result Variability

#### Impact of Inconsistent Anatomical Definitions

One of the critical findings from this review is that the discrepancy between anatomical definitions of the AT and triceps surae tissues seems to be a key source of variability associated with the reported strain values. Here, the use of different definitions for the tendon aponeurosis (Fig. 2) clearly leads to the measurement of different structural components of the AT and hence results in different strain values. For example, the fibrous tissue that attaches to the soleus above the soleus MTJ would be considered the proximal section of the AT when using convention “a”, a mid-section of AT when using convention “b”, and the distal aponeurosis when using convention “c”. Similarly, the disagreement over the observation that within the MG sub-tendon the FT exhibits larger strains than the aponeurosis [63, 67, 83, 93], can be explained by a difference in anatomical definitions. These four papers used the convention “c”, whereas the two authors reporting the opposite result [37, 99] used convention “b”. To avoid comparing strain results that pertain to different structures, we therefore recommend to explicitly define the AT and aponeuroses in all future studies, as well as using the most common convention “c” where possible.

#### Impact of the Metric Choice

The use of strain as a metric to assess tissue functional deformation can lead to several problems. True strain is calculated with respect to tendon slack length—the greatest length before which passive tension forces rapidly increase [124]. However, the associated triceps surae muscles’ slack lengths are known to be dependent on joint angles: 20° ankle plantarflexion for the MG, 15° for the LG, and 2° for the soleus when the knee is extended [139], suggesting that AT sub-tendons have individual slack lengths. Similarly, the FT has been shown to become slack when the ankle joint passively reaches approximately 44° plantarflexion [140]. Importantly, these results suggest that the FT is nearly always elongated, and that AT strain results are most likely underestimated when common reference poses are selected: heel strike, toe off, standing, or at a given ground reaction force level. Additionally, in cases where the slack length is unknown, the reported metric necessarily becomes relative elongation rather than strain, such that the reported values are relative to the chosen reference length, rather than an absolute known value. In fact, most studies that report AT strain actually disclose relative elongations, normally as peak values along the longitudinal (superior–inferior) axis. Since the experimental and clinical assessment of true tendon slack length remains extremely challenging, it is therefore critical that authors report their AT reference lengths in order to allow at least a comparison of relative elongation between studies. Moreover, as far as possible, studies should report the conditions under which the reference length was established, including knee and ankle flexion angles. Although reporting other metrics such as elongation or displacement allows investigators to circumvent assessing slack length, any comparison against strain then becomes misleading. For example, during submaximal contractions, the LG and the MG sub-tendons exhibit a similar elongation [81], but the shorter slack length of the MG sub-tendon produces a greater strain than in the LG sub-tendon.

#### Impact of Measurement Methods

Each measurement method has limitations that inherently affect AT strain measurements and hence study comparisons (Additional file 1: Online Resource S1, Strain Measurement Methods section). The most commonly used assessment technique, US, is limited by fibre sliding and rotations, which confound the imaging plane and thus add measurement uncertainty, particularly during dynamic activities. Here, twisting of the MG, LG, and soleus sub-tendon fibres further challenges the ability of this 2D technique to track the structure of interest. Similarly, motion capture techniques are subject to skin tissue artefact, which is known to lead to considerable errors [106]. While some motion capture results during running and walking were consistent with US for strains in the MG sub-tendon [112, 123], the strain in the FT was underestimated by a factor of two [65]. Moreover, the strain in the MG sub-tendon during running has been reported to be as low as 1% [117]. As a result, there is clearly still a critical need to validate results achieved through motion capture and computational modelling studies. While MRI measurements allow excellent visualisation and hence access to soft tissue elongation patterns, the technique is limited to activities that are performed within the restricted field of view of the MRI bore, such as submaximal contraction. However, during such activities, the longitudinal strain ratio between the FT and aponeurosis is known to change [67, 86], as well as the relative force contributions of the MG and LG [81]. Therefore, caution should be taken in extrapolating submaximal contraction strain patterns from MRI to functionally loaded activities with greater contraction levels. Other techniques such as the radiographic assessment of tantalum beads inserted into the AT [141] could present interesting options for further research into the tissue’s functional behaviour, particularly if combined with moving fluoroscopy to access dynamic activities.

#### Impact of Data Post-processing

Importantly, after measurement, different post-processing approaches to calculate AT length (e.g. the straight line model [142], extraction of AT length from MTU length [132], correction for joint rotations [36], or the mitigation of soft tissue artefact when using motion capture or ultrasound [106]) all yield distinct results. Here, common assumptions such as the AT having a linear connection from the MTJ to the calcaneus are challenged by muscle bulging or the tendon becoming slack [143] and can hence significantly underestimate the reference length and introduce error into strain estimates [128].

### Variability of Strain Results During Dynamic Activities

#### Different Sub-structures Present Inhomogeneous Strain Patterns

The measured and estimated strains in the AT were highly inhomogeneous, ranging from zero (and even negative) in the MG aponeurosis to magnitudes of up to 16% in the FT during running. Compared to direct strain measurements in other animals (e.g. up to approximately 4.15% strain in pony superficial digital flexor tendon during trotting [144] and between 1 and 2% in the sheep forelimb lateral digital extensor during trotting [145]), these estimates of strain in the human AT are generally high. One of the most interesting aspects was the clear differences in AT strain patterns between the different sub-structures. In fact, it is likely that the special anatomical and structural make-up of the AT accounts for the strong strain inhomogeneity (Fig. 4), including the shortening of the aponeurosis at the soleus MTJ observed in several studies [57, 86, 100] (Fig. 5). Here, it seems likely that localised contraction of the soleus muscle, combined with widening of the muscle belly, both occurring at the point of MTJ attachment, could indeed result in local negative longitudinal strains, even though the more global structure is being elongated. One possible contribution to this observed behaviour is the fact that aponeuroses are not mechanically in series with tendons or the contractile fibres of the muscles; rather their elongation or shortening depends on the shear and pressure forces of the muscle as well as the stiffness of the muscle itself, which varies with muscle force. Thus, elongation or strains in aponeuroses cannot not provide information about the properties (stiffness, elastic modulus) of the aponeurosis since the loading conditions within the aponeurosis cannot yet be measured. However, forces in the aponeuroses are known to vary with length along the structure, with the highest forces occurring at the distal extremity (near the AT insertion) and progressively decreasing towards the proximal end (where the forces theoretically reduce to zero) [146]. Thus, local elongations of the aponeuroses are influenced by the force gradients along the length of the aponeurosis. As a result, the contribution of the aponeuroses to AT elastic energy storage is controversially discussed [64, 90, 147]. It is these arguments that have prompted some authors to suggest that assumptions regarding the aponeuroses should be entirely reconsidered [148]: the inclusion of aponeuroses as series elastic elements in MTU models tends to overestimate the amount of stored and released mechanical energy.

#### Consensus and Ongoing Uncertainties About the Strain Patterns During In Vivo Activities

During walking and running, the MG and LG sub-tendons undergo similar elongation patterns, which are consistent with MVC measurements [37, 38], but of a lower magnitude than the FT [103, 139]. As a result, the MG and LG aponeuroses (according to convention “c”, Fig. 3) must strain less than the FT in the longitudinal plane, which has been observed in vivo [67]. However, both positive and negative transverse strains have been observed for the aponeuroses. Positive transverse strains plausibly result from the pressure exerted by the muscles bulging during contractions [41], but negative strains have also been explained by the positive Poisson’s coefficient of tendons, where positive longitudinal strains lead to negative transverse strains [12]. The strain behaviour of the aponeurosis is inconsistent among studies and difficult to measure because its mechanical properties depend on the kinematics, the location of measurement, and the muscle activation level. Here, one interesting consideration is that muscle length changes can be achieved by either shortening/lengthening or by fibre rotation (change in pennation angle). As a result, length changes in a muscle can occur without appreciable length changes in the aponeuroses due to their medial and lateral fibres gliding relative to one another.

**Fig. 6:**
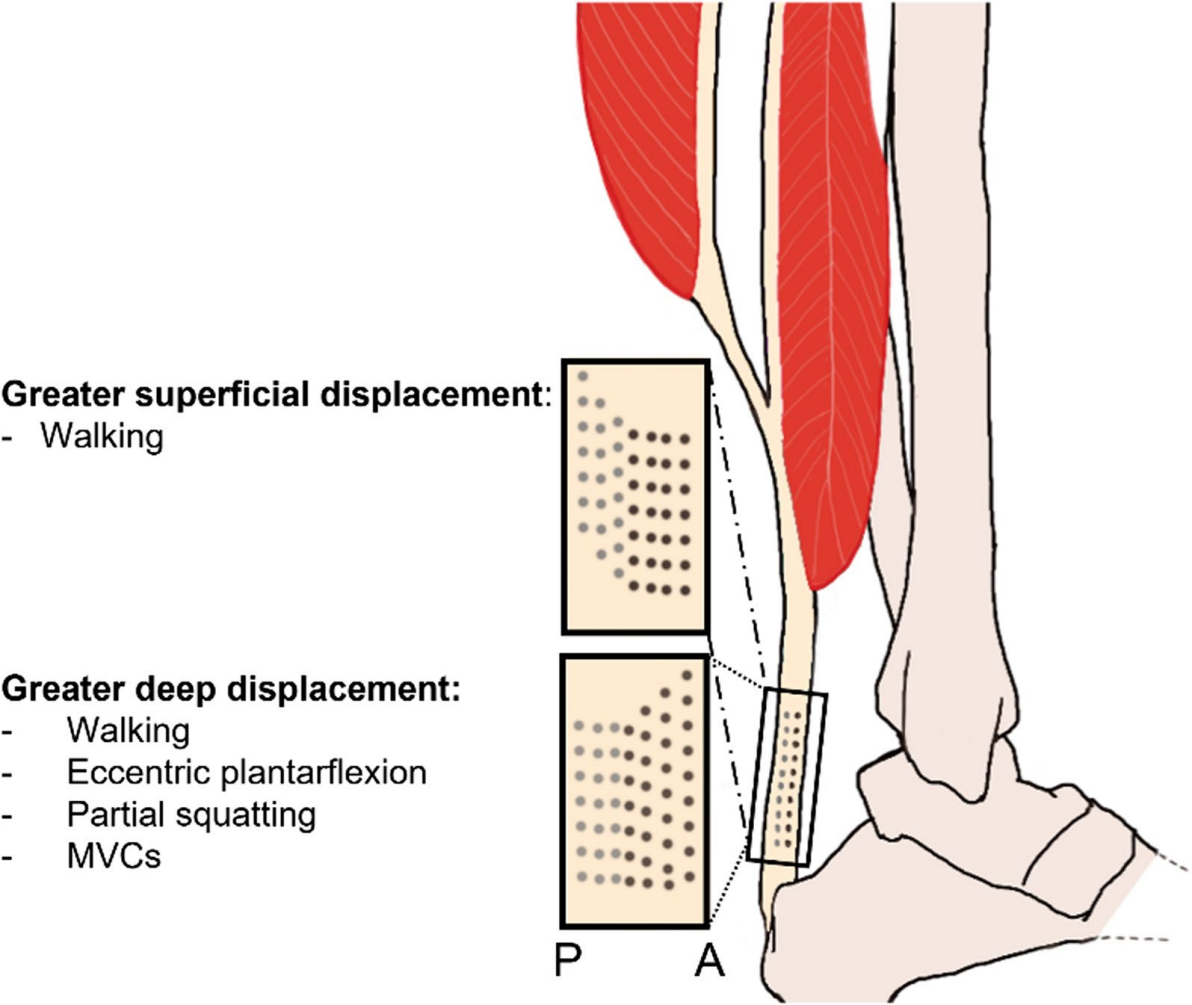
Sagittal cut of the free tendon showing the difference in longitudinal elongation between superficiaol and deep layers during several activities. The grey dots represent the superficial/gastrocnemius layers, and the black dots represent the deep/soleus layer. A and P stand for the anterior and posterior directions along the sagittal plane

During walking, the three MTUs behave differently: the MG and LG muscle fascicles remain isometric or slightly shorten during the late stance phase, whereas the soleus muscle fascicles lengthen [43, 105]. Interestingly, increasing walking speed predominantly increases the activation of the gastrocnemius muscles [149] in a process that possibly leverages energy from the knee due to their two-joint nature. The strain pattern of the AT during walking resembles the energy storage patterns observed for running with the stretch during stance phase and the catapult recoil and release of energy during push-off, albeit happening more slowly because of the longer stance phase [62]. Due to methodological limitations, the distribution of local longitudinal strains within the MG or LG sub-tendons has not been well studied outside of MVCs. Some studies have, however, measured displacements within the FT during walking. Here, similar patterns to those seen during MVCs, where the deep FT elongates more than the superficial layer, have been measured [37, 63, 104], which has also been found in passive and eccentric plantarflexions [92, 150, 151] and in partial squatting [152, 153] (Fig. 6). The reasons might be that the superficial region originating from the MG is more compliant, or that tendinous fascicle sliding confounds ultrasound measurements [150]. The observation is not undisputed, however, since superficial layers of the AT elongating more than the deeper layers during walking have also been reported [102, 103].

#### Potential Explanations for Unexplained Strain Patterns

What could explain the variability between MVC and walking in the change of sub-tendon patterns? Kinetic and kinematic differences could provide an answer. First, constraining the foot seems to have an influence: strain results during plantarflexion vary if the foot is constrained and unexpected shortenings have been measured when unconstrained [143]. Knee and ankle flexion angles also clearly influence tendon slack length and 3D AT moment arm [154], which can also increase with larger muscle contraction as the muscles get thicker [155]. Importantly, the knee angle also influences fascicle length of the biarticular MG and LG muscles. Indeed, during eccentric ankle plantarflexions, when the knee is flexed, the strain within the soleus sub-tendon is altered but not within the MG and LG (superficial) sub-tendons [150]. Given that EMG levels of the triceps surae muscles differ considerably between activities [154] and that their relative activation level is modulated by contraction level [96], this might lead to a preferential loading of the soleus when the shortened gastrocnemius fascicles might not be able to generate force at the very bottom of the force/length curve [37, 150]. Respective force optima are therefore likely to occur at different stages of the gait cycle, explaining why the soleus is thought to primarily deal with body weight support, whereas the gastrocnemius mostly contributes to propulsion [103]. Here, the locations of the gastrocnemius and the soleus attachment points likely play key roles in the local mechanics at different stages of the gait cycle. Due to their orientation, the soleus muscle generates a force vector axial with the tibia, whereas the gastrocnemius muscles create a vector pointing slightly backwards, more suitable to efficient propulsion. Finally, during walking, energy is conserved by positive muscle work throughout the lower limb [146], enabling the gastrocnemius muscles to leverage and transfer energy from the knee extensors during unconstrained movement due to their two-joint nature. These kinematic and kinetic boundary conditions might explain why different strain patterns are observed within the FT between MVCs and walking.

The role of connective tissues and energy transfer within the triceps surae MTUs is unclear. Myofascial force transmission between muscles and tendinous connective tissues seems to distribute force within the AT [156]. The extent of these transmissions depends on muscle groups, muscle lengths, and activation levels [156]. Furthermore, the extent of tendon fascicle sliding needs to be quantified, as comparative studies indicate that this twisted rope-like behaviour might help store energy in tendons [103].

## Conclusions

From the complex interactions between AT anatomy, including its sub-tendons and aponeuroses, as well as knee and ankle joint flexion angles and uni- and biarticular muscular structures and their loading conditions, it has become clear that numerous questions remain regarding AT function in vivo. The addition of AT material properties, which is beyond the scope of this review, adds further uncertainty. However, what is clear is that the AT can no longer be considered a single homogeneous structure, but rather needs to be investigated as multiple sub-structures with complex interactions. In order to enhance training and performance, understand injury mechanisms, as well as improve repair and rehabilitation protocols, further investigation into the individual AT sub-structures and their interactions is clearly needed. To ensure that studies can be universally compared, we strongly recommend that the locations of measurements are standardised with respect to a consistent definition of the anatomical sub-structures, together with unambiguous reporting of the reference conditions.

## Supplementary Information

**Additional file 1.** Online Resource S1.

**Additional file 2.** Online Resource S2.

## Data Availability

All data generated or analysed during this study are included in the published article and its supplementary information files. Additionally, any inquiry regarding the data can be asked to the corresponding author.

## Abbreviations

A-P: Antero-posterior
AT: Achilles tendon
CSA: Cross-sectional area
FT: Free tendon
LG: Lateral gastrocnemius
MG: Medial gastrocnemius
M-L: Medio-lateral
MRI: Magnetic resonance imaging
MTJ: Muscle-tendon junction
MTU: Muscle-tendon unit
MVC: Maximal voluntary contraction
OTJ: Osteo-tendinous junction
S-I: Superior–inferior
VC: Voluntary contraction
