# Supplementary material for: In Vivo Strain Patterns in the Achilles Tendon During Dynamic Activities: A Comprehensive Survey of the Literature
Source: Sports Med Open. 2023 Jul 19;9:60. doi: 10.1186/s40798-023-00604-5 (PMC10356630; doi:10.1186/s40798-023-00604-5)
Supplement: Supplementary file 1 — Additional file 1. Online Resource S1. [file 40798_2023_604_MOESM1_ESM.docx]

# In Vivo Strain Patterns in the Achilles Tendon During Dynamic Activities: A Comprehensive Survey of the Literature

Naomi C. Adam^1^, Colin R. Smith^1^, Walter Herzog^2^, Andrew A. Amis^3^, Adamantios Arampatzis^4^,

William R. Taylor^1^

^1^ Institute for Biomechanics, ETH Zürich, Switzerland

^2^ Human Performance Laboratory, Faculty of Kinesiology, The University of Calgary, Canada

^3^ Department of Mechanical Engineering, Imperial College London, United Kingdom

^4^ Department of Training and Movement Sciences, Humboldt‐Universität zu Berlin, and Berlin School of Movement Science, Berlin, Germany

_________________________

**Corresponding Author**

Prof. Dr. William R. Taylor

ETH Zürich

Institute for Biomechanics

Leopold-Ruzicka-Weg 4

8093 Zürich

Switzerland

Email : bt@ethz.ch

**Online Resource 1**

**Content**

- The Techniques for Strain Measurement (Online Resource 1)
- The quantitative and qualitative data stored in tables in an excel file (Online Resource 2)
- The methods and plots of available data for walking, running, jumping, and landing (Online Resource 1)

**Techniques for Strain Measurement**

Techniques to investigate AT strain are generally based on ultrasound, MRI or a combination of motion capture and ultrasound/musculoskeletal modelling. Ultrasound is the most frequently used technique to measure AT strain because it is non-invasive, low-cost, easy to implement in most clinical or laboratory settings, and capable of imaging the AT throughout dynamic movements. The equipment consists of a monitor and a cart wired to a hand-held transducer; this flexibility allows the recording of dynamic activities such as walking, running, and jumping by strapping the transducer to the calf. The working principle behind ultrasound imaging relies on propagating ultrasonic waves into the tissue of interest and recording their reflection [27]. Investigators have used both the unprocessed radiofrequency (RF) signals or more commonly processed B-mode images, where a two-dimensional image is generated by shading pixels based on the amplitude of the reflected wave (filtered RF signal). From such ultrasound images, two primary techniques have then been used to extract AT strain: MTJ tracking and speckle tracking (digital image correlation). MTJ tracking measures the relative distance between two anatomical landmarks throughout an activity and then computes strain based on its slack length [27, 150]. A common use of this technique is to track the displacement between the calcaneus OTJ and the medial gastrocnemius MTJ. Here, the OTJ is commonly outside the field of view of standard sized transducers, so prior to the experiment, ultrasound is used to identify the OTJ, and the location is marked on the skin. Then a motion capture marker is placed at this landmark and a marker triad attached to the ultrasound probe (viewing the MTJ) allows relative navigation and hence a common coordinate system. However, skin motion artifact can introduce significant error as the elongation of the stiff AT is often only a few millimetres [27], but the relative movement of the probe can be centimetres. The second ultrasound technique, speckle tracking, has been applied to both B mode and RF images [150]. Here, the movement of light and dark spots in the ultrasound images is tracked over time using automated algorithms such that the relative distance between spots on the AT tissue structure can be monitored [27]. While any change in the relative distance between spots is considered to be a metric of elongation, a key limitation of this method is that images are confounded by out of plane movement, such as tendon fibres rotating or inter-fibre sliding. Freehand 3D ultrasound overcomes this limitation by scanning multiple regions to stack 2D images into a 3D structure [83]. Here, however, the subject has to remain absolutely static during the 10-15s scan time, which limits the measurements to non-dynamic activities e.g. passive and sub-maximal isometric contractions.

MRI enables static non-invasive imaging of the AT, but this technique remains less frequently used because it is expensive, limits the activities that can be performed within the MRI bore, and has a lower spatial resolution than ultrasound. This technique relies on accurate identification the attachment sites of the AT in the MRI slices [27, 96]. Here, multiple poses must be taken to calculate a displacement within the desired region of interest. Tissue strain is then computed by dividing by the slack length.

The third technique to quantify AT strains couples motion analysis measurements and musculoskeletal modelling. This indirect approach offers the greatest flexibility and accessibility to investigate unconstrained dynamic movements but is potentially subject to artefacts. Here, AT strain can be estimated via two methods, either using motion capture alone or coupled with other indirect techniques: 1) AT displacements are approximated based on the relative motion of reflective markers placed on the skin over the calcaneus and the insertion of the sub-tendon of choice. 2) Kinematics and kinetics are measured during the activity using marker sets and force platforms. AT strain is then calculated by dividing the AT force, obtained through inverse dynamics, by the Young’s Modulus and the cross-sectional area of the free tendon (which itself varies according to measurement location). These two parameters can either be measured by ultrasound or MRI [133], or extracted from the literature [132]. Another recent possibility is to use the AT force as a boundary condition for a personalized AT finite element model made with 3D ultrasound that can calculate the principal components of strain and generate a subject specific strain pattern [112]. The limitations of these approaches include experimental sources of error such as skin motion artifact and musculoskeletal modelling assumptions involving tissue properties, geometries, and muscle co-contraction.

**Graphical representation of patterns during activities**

Purpose: The goal of the graphical representation was to display the pattern evolution with respect to time during functional activities. These graphs are meant to illustrate the timing of pattern change, but the magnitude of the values cannot reliably be compared between authors due to the difference in protocols.

Methods: AT patterns were plotted against time (normalized to 100% of an activity cycle) for walking, running, jumping, and landing for each study where data were available. The sub-structures investigated by the authors were indicated in the legend of the graph. The data points were digitized from figures using WebPlotDigitizer v4.2. Given that very few graphs offered strain data in the literature, we used elongation as a metric instead. Elongation refers to the difference between the tendon instantaneous length and a reference length, where the reference length has been set at zero for the graphs for this application. When length changes were reported, the values were normalized by subtracting the minimal value to all data points. Whenever The data were then interpolated with the ‘spline’ option of the MATLAB interp1 function and plotted.

Activities

1. Walking

**Fig S1**: Elongation profile of the medial gastrocnemius (MG) lateral gastrocnemius (LG), and soleus (SOL) sub-tendon during walking. The vertical black line represents the approximate beginning of the swing phase. One author only reported data points for the stance phase.


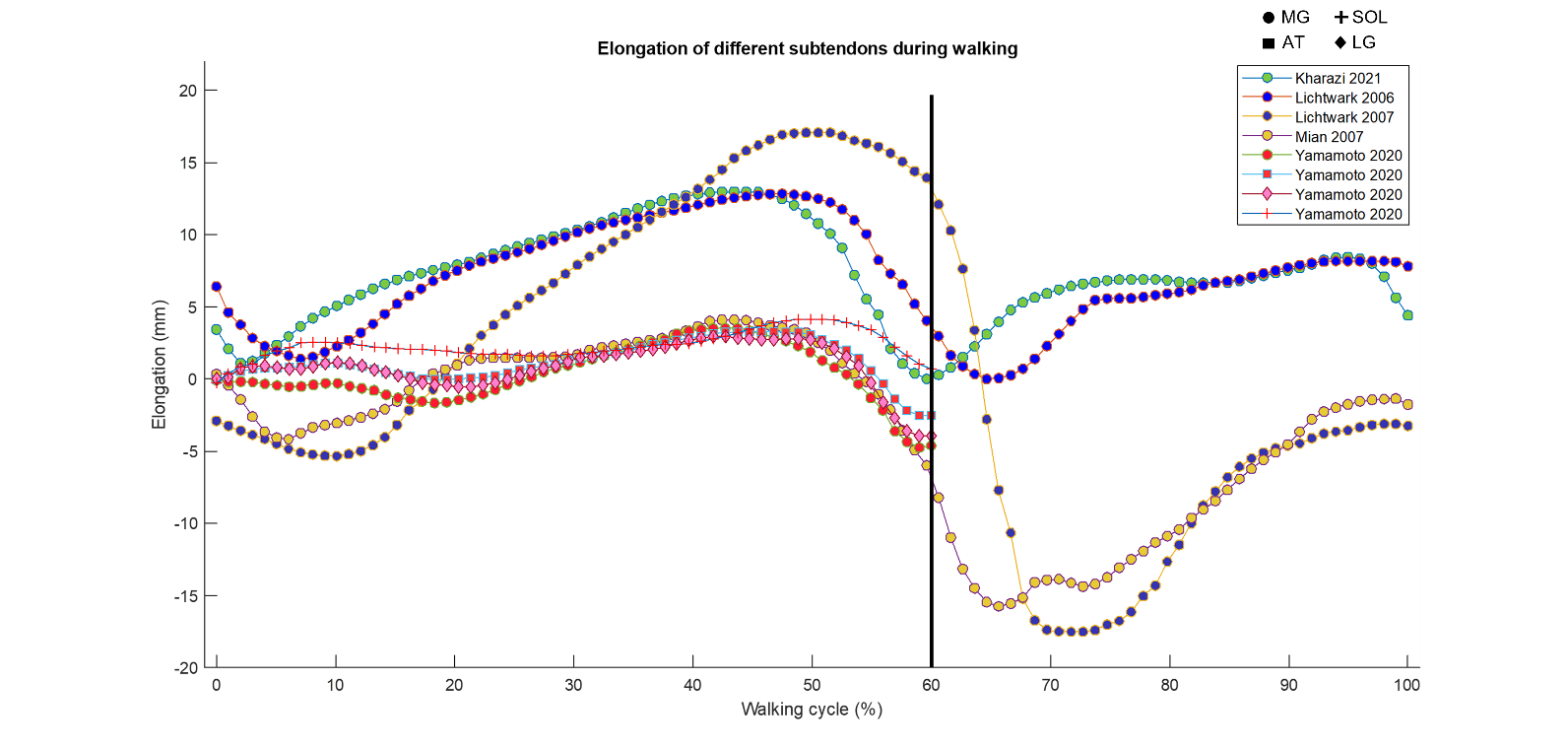


1. Running

**Fig S2**: Elongation profile of medial gastrocnemius (MG) and soleus (SOL) sub-tendons during running. The vertical line represents the approximate beginning of the swing phase. Two authors only reported data points for the stance phase.


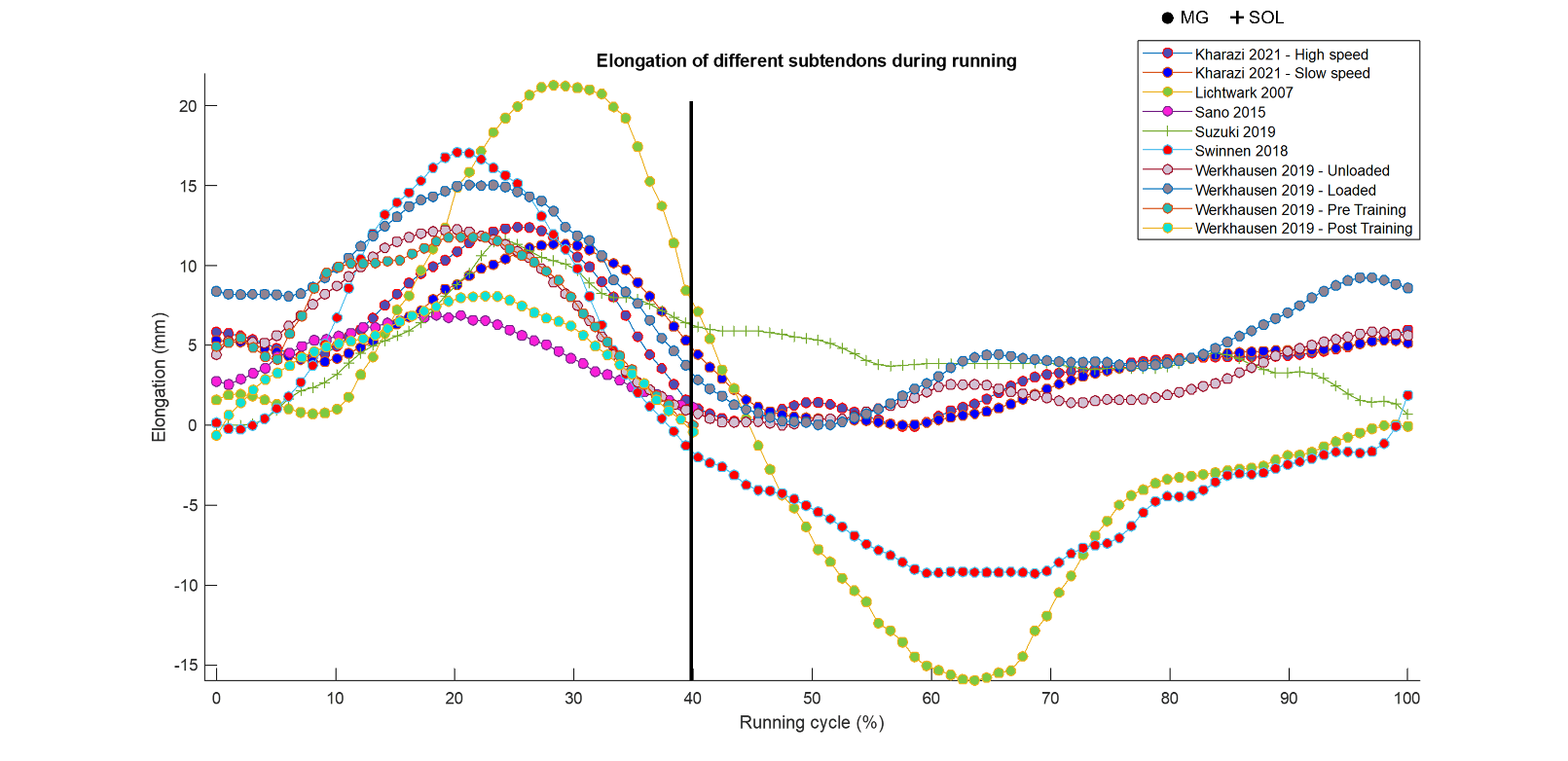


1. Jumping

**Fig S3:** Elongation of the medial gastrocnemius sub-tendon during different jumping styles.


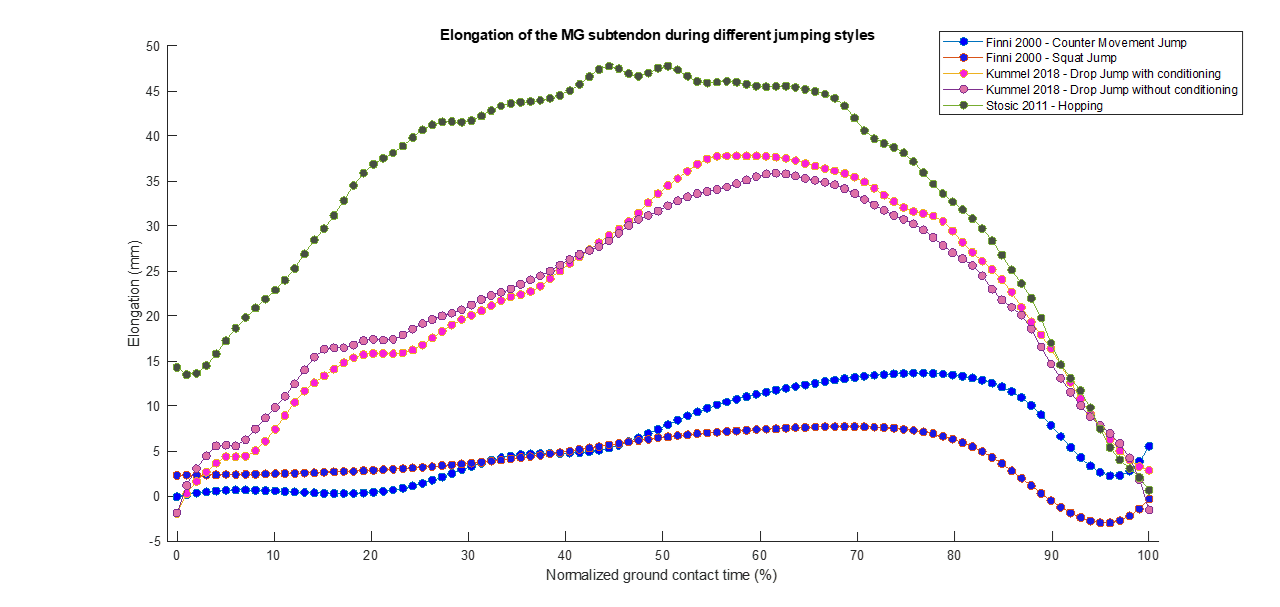


1. Landing


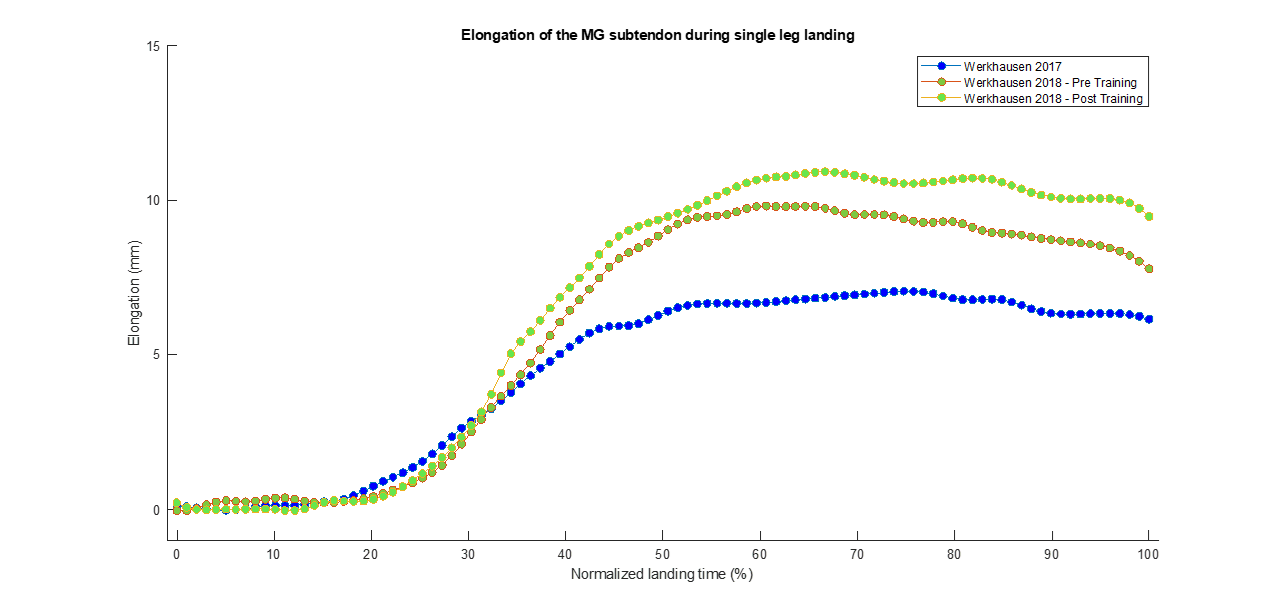


**Fig S4:** Elongation of the medial gastrocnemius sub-tendon during single leg landing.

**References (Consistent with main manuscript)**

27. Zhang Q, Adam NC, Hosseini Nasab SH, Taylor WR, Smith CR. Techniques for In Vivo Measurement of Ligament and Tendon Strain: A Review. Ann Biomed Eng. 2020.

83. Farris DJ, Trewartha G, McGuigan MP, Lichtwark GA. Differential strain patterns of the human Achilles tendon determined in vivo with freehand three-dimensional ultrasound imaging. J Exp Biol. 2013;216(Pt 4):594-600.

96. Reeves ND, Cooper G. Is human Achilles tendon deformation greater in regions where cross-sectional area is smaller? J Exp Biol. 2017;220(Pt 9):1634-42.

112. Pizzolato C, Shim VB, Lloyd DG, Devaprakash D, Obst SJ, Newsham-West R, et al. Targeted Achilles Tendon Training and Rehabilitation Using Personalized and Real-Time Multiscale Models of the Neuromusculoskeletal System. Front Bioeng Biotechnol. 2020;8:878.

132. Finni T, Komi PV, Lepola V. In vivo human triceps surae and quadriceps femoris muscle function in a squat jump and counter movement jump. Eur J Appl Physiol. 2000;83(4 -5):416-26.

133. Firminger CR, Bruce OL, Wannop JW, Stefanyshyn DJ, Edwards WB. Effect of Shoe and Surface Stiffness on Lower Limb Tendon Strain in Jumping. Med Sci Sports Exerc. 2019;51(9):1895-903.

150. Slane LC, Thelen DG. Non-uniform displacements within the Achilles tendon observed during passive and eccentric loading. J Biomech. 2014;47(12):2831-5.
